# Assessing the contextual effect of community in the utilization of postnatal care services in Ghana

**DOI:** 10.1186/s12913-020-06028-1

**Published:** 2021-01-07

**Authors:** Emmanuel Dankwah, Cindy Feng, Shelley Kirychuck, Wu Zeng, Rein Lepnurm, Marwa Farag

**Affiliations:** 1 School of Public Health, University of Saskatchewan, 104 Clinic Place, Saskatoon, SK S7N 2Z4 Canada; 2 Department of Community Health and Epidemiology, Faculty of Medicine, Dalhousie University, Centre for Clinical Research, 5790 University Ave., Halifax, NS B3H 1V7 Canada; 3 Department of Medicine, College of Medicine, Canadian Centre for Health and Safety in Agriculture (CCHSA), 104 Clinic Place, Saskatoon, SK S7N 2Z4 Canada; 4 School of Nursing & Health Studies, Georgetown University, 3700 Reservoir Rd, Washington, DC 20007 USA; 5 School of Public Administration and Development Economics, Doha Institute for Graduate Studies, Al Tarfa Street, Zone 70, Doha, Qatar

**Keywords:** Postnatal care, Contextual effect, Community, Utilization

## Abstract

**Background:**

Inequalities in the use of postnatal care services (PNC) in Ghana have been linked to poor maternal and neonatal health outcomes. This has ignited a genuine concern that PNC interventions with a focus on influencing solely individual-level risk factors do not achieve the desired results. This study aimed to examine the community-level effect on the utilization of postnatal care services. Specifically, the research explored clusters of non-utilization of PNC services as well as the effect of community-level factors on the utilization of PNC services, with the aim of informing equity-oriented policies and initiatives.

**Methods:**

The 2014 Ghana Demographic and Health Survey GDHS dataset was used in this study. Two statistical methods were used to analyze the data; spatial scan statistics were used to identify hotspots of non-use of PNC services and second two-level mixed logistic regression modeling was used to determine community-level factors associated with PNC services usage.

**Results:**

This study found non-use of PNC services to be especially concentrated among communities in the Northern region of Ghana. Also, the analyses revealed that community poverty level, as well as community secondary or higher education level, were significantly associated with the utilization of PNC services, independent of individual-level factors. In fact, this study identified that a woman dwelling in a community with a higher concentration of poor women is less likely to utilize of PNC services than those living in communities with a lower concentration of poor women (Adjusted odds ratio (AOR) = 0.60, 95%CI: 0.44–0.81). Finally, 24.0% of the heterogeneity in PNC services utilization was attributable to unobserved community variability.

**Conclusion:**

The findings of this study indicate that community-level factors have an influence on women’s health-seeking behavior. Community-level factors should be taken into consideration for planning and resource allocation purposes to reduce maternal health inequities. Also, high-risk communities of non-use of obstetric services were identified in this study which highlights the need to formulate community-specific strategies that can substantially shift post-natal use in a direction leading to universal coverage.

## Background

The post-natal period is a critical stage in the obstetric cycle, especially, the first 24 h and early days following childbirth [1, 2]. Women and babies need special attention during this period because most deaths occur in that time [3]. Moreover, studies have found that adequate care during the post-partum period is vital for maternal and child survival, especially in poorer regions of the world with high maternal and neonatal mortalities [4, 5]. Researchers contend that achieving a post-natal care (PNC) services utilization rate of 90% in Africa could save between 10 to 27% of neonatal deaths [2].

**Fig. 1:**
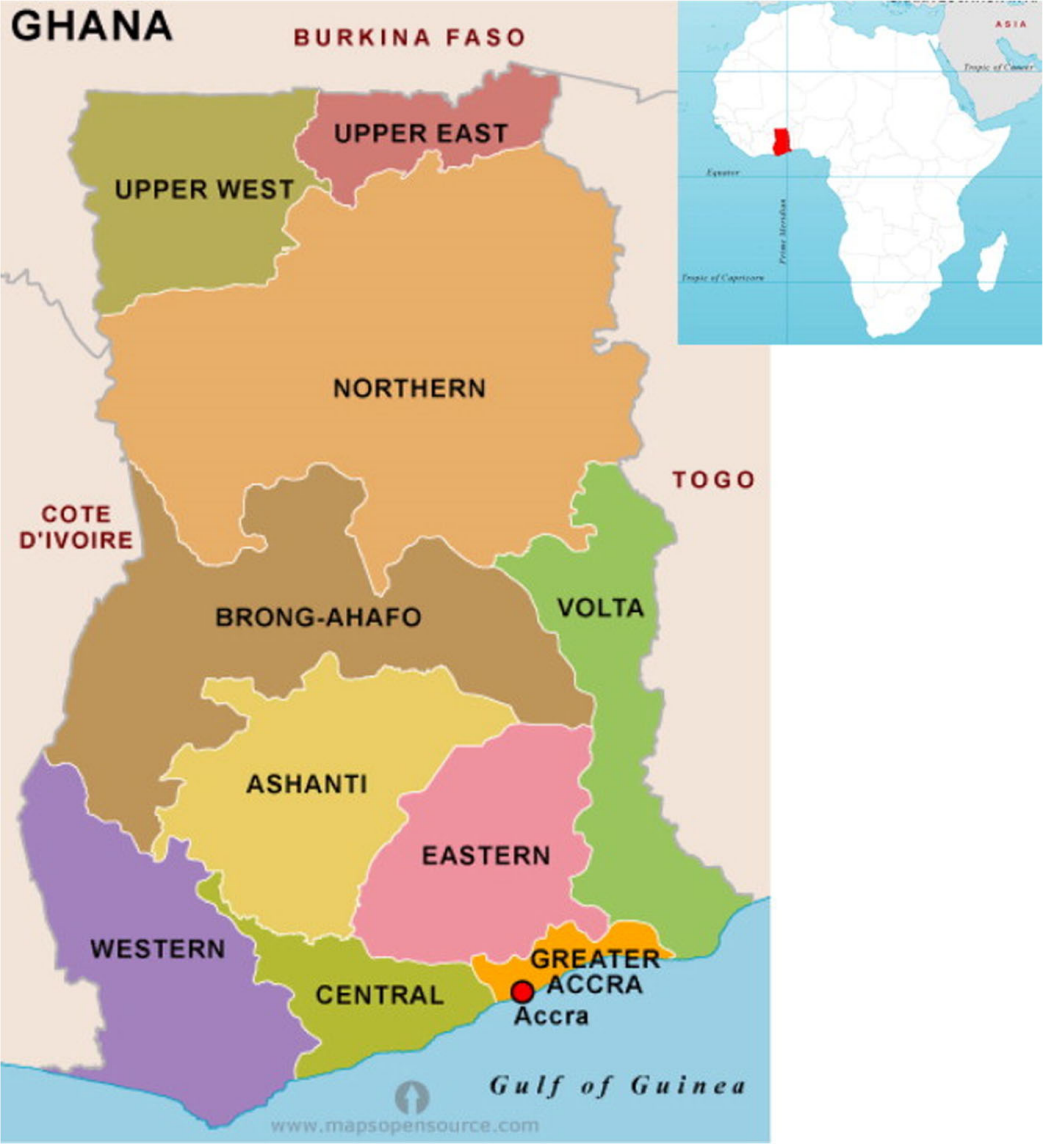
ap of Ghana and its location in Africa. Source: http://www.mapsopensource.com

Despite the benefits derived from PNC services, a large proportion of sub-Saharan Africa mothers and babies especially those that delivered outside health facility do not use post-natal services [6]. Ghana is a western African country that spans a land area of 238,535 km^2^ [7] and shares boundary with Burkina Faso to the north, Cote d’Ivoire to the west, Togo to the east and the Gulf of Guinea on the south (Fig. 1). Ghana had 10 administrative regions during the 2014 Ghana Demographic and Health Survey (GDHS) namely Western, Central, Greater-Accra, Volta, Eastern, Ashanti, Brong Ahafo, Northern, Upper East and Upper West (Fig. 1). The situation is not different in Ghana, a nation with a fertility rate of 4 and approximately 76% livebirths from all pregnancies [7]. A critical mass of mothers and newborns lack needed PNC services [7, 8]. A previous study conducted in one administrative region of Ghana reported that PNC services covered 43.8% of mothers [8] whereas another study revealed that 56% of neonates immediately received PNC services within 48 h after their birth [9] in Ghana. Such underutilization and discrepancies in the usage of postdelivery services exposes mother and newborn to higher risk of morbidity and mortality [10], as well as undermining healthy behaviors and practices such as exclusive breastfeeding and uptake of family planning [1].

It is manifestly clear that identifying determinants of PNC service utilization are necessary to guide public health interventions. Most studies on the usage of PNC services are concentrated on identifying individual-level factors [11–15]. Although evidence exists at the individual-level that PNC services utilization is associated with socioeconomic predictors [13, 16–19], there is a general concern that PNC interventions focused solely on influencing individual-level risk factors do not achieve the desired results. For this reason, recent studies considered community-level effects [20, 21], but the results were mixed. For instance, Worku et al. [22] found no significant area-level effects on the use of PNC services. Some multilevel studies in Tanzania [23], Nigeria [24], Zambia [25], Kenya [26] and India [27, 28] reported that a community-level effect strongly predicted PNC services use, after controlling for individual-level predictors. In addition, these previous studies estimated different community-level effects of communities on the utilization of PNC services; for example Mohan et al. [23] and Solanke et al. [29] reported that 12 and 37.2% unexplained community variance respectively accounted for the variability in PNC services use. Many methodological limitations accompanied these contradictory findings including the use of a limited number of independent variables and inadequate adjustment for confounding.

Likewise, inequalities in the use of PNC services across communities have been observed in Ghana [7]. However, research about the utilization of PNC services in Ghana is scanty [8, 30]. In addition, most of the studies were restricted to a specific geographical area of the country which seriously affects the potential for extrapolation of findings to the entire population [8, 30]. A thorough literature review indicated that community-level effects on variation in the use of PNC services in Ghana remain uncertain. The review showed that the majority of studies conducted in Ghana used only individual-level data [12]. Hence, there is a dearth of evidence measuring the association between community-level risk factors and the utilization of PNC services specific to Ghana. This study intends to assess (1) the community-level effect on the utilization of PNC services in Ghana using a multilevel regression model with a logit link function and (2) explore the spatial pattern of non-use of PNC services across Ghanaian communities. The findings from this study could be used to inform equity-based interventions to improve the use of PNC services across communities in Ghana and other low-income countries.

## Methods

### Data source: Ghana demographic health survey (2014- GDHS)

This research used the 2014 Ghana Demographic and Health Survey (GDHS) dataset. The Demographic and Health Survey (DHS) program has a long-standing reputation for the collection, processing and sharing of population and health information in over 90 low-and lower-middle-income countries, including Ghana. The 2014 GDHS was conducted in Ghana by Ghana Statistical Services, Ghana Health Services and the National Public Health Laboratory with technical support from the Inner City Fund (ICF) international.

**Fig. 2:**
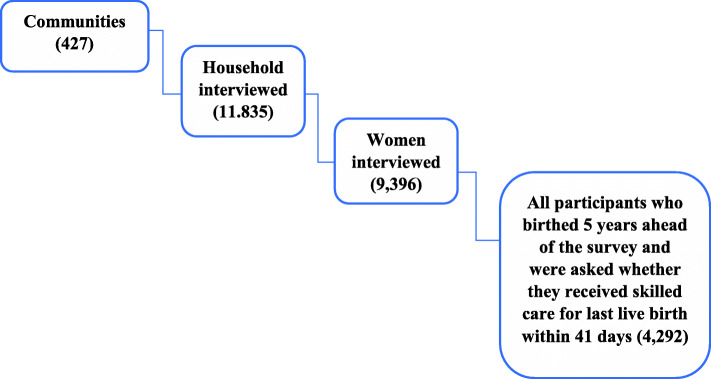
Hierarchical structure of the 2014 GDHS data

According to the 2014 GDHS report, the participants of this survey were sampled using a two-stage sampling technique. The sampling process started with the random selection of 427 clusters (hereinafter community) that were representative both at the national (10 regions) and residence (urban and rural) level using the 2010 Ghana population and housing census sampling frame as a guide. Furthermore, using systematic sampling technique about 30 houses were chosen from each cluster amounting to 12,831. Among the selected houses, 12,010 were occupied and the remaining houses were vacant. A response rate of 99% was achieved for successfully interviewing 11,835housholds out of the 12,010 households. Overall, 9396 women aged 15–49 years were interviewed after consenting to participate out of the 9656 women that were eligible for the survey in the interviewed households representing 97% response rate [7]. This nationwide survey captured information including women’s reproductive health, health-seeking behavior, socioeconomic, and demographic background as well as geo-reference data. The Global Positioning System (GPS) data were collected for all the 427 communities in Ghana. Out of the 9396 interviewed women, a subgroup of 4292 respondents gave birth in the previous 5 years before the survey and were asked whether they received skilled care 41 days after live delivery (Fig. 2). All the eligible participants (4292) responded to the interview question, which constituted the study sample for this research. More detailed information on the sampling procedure and data is published in the Ghana Demographic and Health Survey of 2014 [7].

### Study variables

The dependent variable was whether the mother received PNC services from a skilled health care provider immediately or within 41 days after delivery. The outcome measure was coded ‘yes or no’ depending on the response to the questionnaire. The questions in the survey regarding PNC services were restricted to last delivery in the past five years hitherto the 2014 GDHS to limit recall bias. The coding structure for the outcome variable has been detailed in Table 1.

**Table 1:** Summary of study variables

Variable name	Variable Description	Coding structure
**Dependent variable**		
Postnatal care usage	Whether postnatal care was received within 41 days after live birth or not	Categorized, 0 = No, 1 = Yes
**Individual-level variables**		
Maternal age	Maternal age in years	Continuous, ranged from 15 to 49 years
Marital status	Current marital status	Categorized, 1 = Single, 2 = Cohabitating, 3 = Widow/divorced/separated, 4 = Married
Religion	Women’s religious affiliation	Categorized, 1 = Traditional/other, 2 = Muslim, 3 = Christian
Ethnicity	Ethnic group of the women	Categorized, 1 = Akan, 2 = Northern tribes, 3 = Ewe, 4 = Ga, 5 = Other
Parity	History of births	Categorized, 1 = One birth, 2 = Two births, 3 = Three or more births
Educational attainment	Highest educational level	Categorized, 0 = No Education, 1 = Primary, 2 = Secondary/ Higher
Wealth status	Wealth status of household	Categorized, 1 = Poor, 2 = Middle, 3 = Rich
Employment status	Working status of the women	Categorized, 1 = Not Working, 2 = Working
**Community-level variables**		
Community problem with distance to health facility	Perceived problem with distance to a health facility when seeking medical care	Categorized, 0 = not a big problem (short distance), 1 = big problem (long distance)
Area of residence	Whether community is rural or urban	Categorized, 0 = Rural, 1 = Urban
Community poverty level	Percentage of women who were poor based on wealth status per community	Categorized, 0 = Low (≤50%), 1 = High (> 50%)
Community educational level	Percentage of women with at least secondary education per community	Continuous, ranged from 0 to 100
Community unemployment level	Percentage of women without work per community	Continuous, ranged from 0 to 81.3

**Fig. 3:**
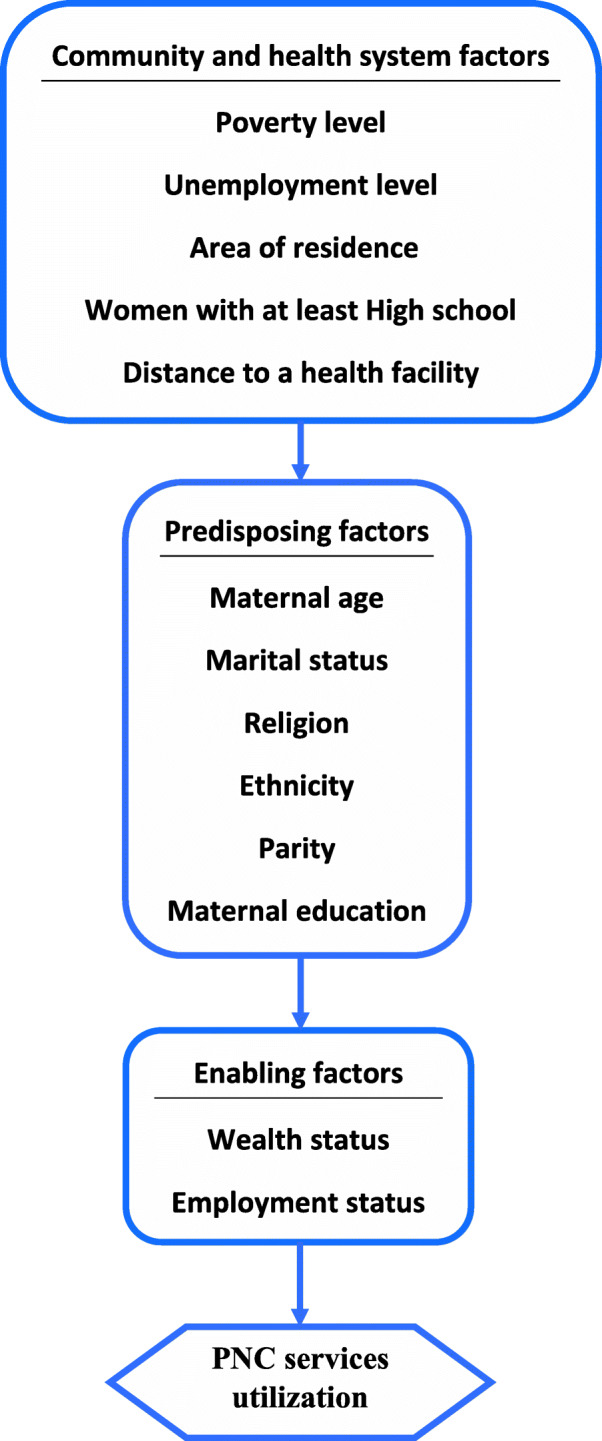
Adapted Andersen’s health utilization model for PNC services utilization

The community-level and individual-level factors employed in this study were chosen using the Andersen health utilization model as displayed in Fig. 3. Community-level factors describe the characteristics of the community while the individual-level factors focus on women’s attributes. Andersen’s behavioral model highlights the health system and community characteristics as well as predisposing and enabling factors as facilitating and inhibitory factors for health care utilization [31, 32].

According to the 2014 GDHS, information about the usual community the women lived in, whether rural or urban was captured and was termed as area of residence in this study. Also, the 2014 GDHS collected information about whether women had an issue with distance to a health facility when seeking medical attention. This survey question captured self-reported information on women’s perceived distance to a health facility in their community and was referred hereinafter in this study as community-level problem with distance to a health facility. According to the 2014 GDHS’s original data file, the variable “problem with distance to facility” was a dummy variable, which was, had two responses either: ‘a big problem’ (indicating longer perceived distance) or ‘not a big problem’ (suggesting shorter perceived distance). This variable was used as a proxy to measure the association of community-level problem with distance to a health facility with the dependent variable as was done in an analogous study in Nigeria [25, 33].

On the other hand, the community poverty level was categorized into two groups as reported in a similar study [34]. The community poverty level variable was created from the wealth index in the survey data. The GDHS wealth index was made from information on possession of household assets and dwelling factors including means of transport, refrigerator, toilet facilities among others [24]. The survey employed Principal component analysis to create wealth index [24] that was categorized into quintiles: poorest, poorer, middle, richer and richest. In this study, the poorest and poorer groups were merged to represent the poor category. The percentage of women who were poor per community was estimated. Community’s level of poverty was coded as high ‘1’ when above 50% otherwise was coded low ‘0’ (Table 1).

The community level of education was generated from women’s responses to the question “highest education attended” in the survey. The survey data categorized the highest education attended into 4 groups namely no education, primary (1–6 years), Secondary (7–12 years) and higher. For this study, the higher education attended variable was created by combining secondary and higher education. The percentage of women with at least secondary education was computed for each community. Also, the community unemployment level was generated from the responses of women on either they were working or not. The survey data created two dummy variables of employment: working ‘1’ and not working ‘0’. The percentage of unemployment per community was calculated. The study variables such as community level of education (*p*-value = 0.67) and community unemployment level (p-value = 0.29) did not fail the linearity test and were examined as a continuous variable. The community-level factors that were analyzed to explain the discrepancies in the utilization of PNC services include the area of residence, community-level problem with distance to a health facility, community poverty level, community education level, and community unemployment level.

Individual-level variables that were studied in this research were maternal age, marital status, religion, ethnicity, parity, education, wealth status, and employment status. As exhibited in Table 1 of this study, maternal age was examined as a continuous variable. Marital status was grouped as single, cohabitating, widow/divorced/separated and married. In terms of religion, this study classified women into traditionalist or other, Muslim, and Christian. Also, ethnicity was classified as Akan, Northern tribes, Ewe, Ga, and other groupings. For this research, the parity of the women was grouped into 1 birth, 2 births, and ≤ 3 births. Women’s highest education level was classified into no education, primary, secondary or higher. This study grouped women’s wealth status into poor, middle, and rich classifications. Finally, women’s employment status was grouped into not working and working. The categorizations of the study predictors were adopted from the literature [35, 36].

### Descriptive statistics

This research employed Chi-square tests to ascertain the differences in the distribution of women across all the categories of the explanatory variables. In this study, proportions and frequencies of postnatal care services use were tabulated according to the hypothesized socio-demographic and economic predictors for women of child-bearing age. Mean, standard deviation, median and interquartile range (IQR) were used for quantitative variables.

### Spatial clustering

This study hypothesized that spatial autocorrelations exist in the use of PNC services across communities. Kulldorff’s spatial scan statistics is a powerful tool to detect spatial autocorrelations based on geographic positioning [37]. This technique was employed in this study to identify local clusters of PNC services across the communities. For analyses, this study used the GDHS spatial data that only allows a set of coordinates per community. A purely spatial analysis was conducted using a discrete binomial model to scan for communities with high rates of non-utilization of PNC services in Ghana. SaTScan technique used in this study hypothesized that the risk of non-use of PNC services was likely different between the inner and outer parts of a circular window. The circular- shape spatial window scan communities to identify areas with a maximum spatial cluster size of 50% of the population at risk. The probability model relied on Monte Carlo simulation with replication of 999 and 50% of the population at risk was considered the maximum size of a spatial cluster [38]. The analyses were conducted using SaTScan software, version 9.6.0. The outputs generated from SaTScan analyses were displayed on Google Map to highlight the spatial patterns of non-use of PNC services.

### Inferential statistics

#### Multilevel mixed regression model

Given the sampling technique and the hierarchical nature of the weighted 2014 GDHS data, a 2-level mixed logistic regression model was specified for the dichotomous outcome [20, 39] using mean-variance adaptive Gauss–Hermite quadrature at an integration point of 12. The components of the model were level one (individuals) nested in level two (communities). This study considers the error in the second level as a random effect to check for disparities in the likelihood of PNC services usage across the communities. The two-level mixed model used is stated below.

Equation 1: Multilevel mixed logistic regression model

where μj ~ N (0, σ^2^_group_); β_0,_ intercept; β_k,_ regression coefficient of the variables; X_ki,_ study predictors; σ^2^_group_, community-level variance.$p\ \left( Yi=1\right)= pi\mathrm{Logit}\ \left(\mathrm{pi}\right)={\beta}_0+{\beta}_1{\mathrm{X}}_1+{\beta}_2{\mathrm{X}}_2\dots +{\beta}_{\mathrm{k}}{\mathrm{X}}_{\mathrm{k}\mathrm{i}}+{\mu}_{\mathrm{group}\left(\mathrm{i}\right)}$

Three models were estimated in this study. A null model was first fitted with no covariates. Second, unconditional mixed logistic regression analyses were conducted between the use of PNC services and each individual-level as well as community-level predictors. Unadjusted odds ratios were generated and correlations with liberal *p*-values of 0.25 or less were selected as candidates for the multivariate 2-level mixed modeling [40]. This unconventional cut-off was used to avoid the elimination of important predictors that could be masked or suppressed by other control variables [40, 41]. Lastly, as proposed by Hosmer and Lemeshow [41], a selection method that manually eliminates insignificant factors was utilized in the final model. This backward technique sequentially removes less relevant characteristics, beginning with the highest p-value and eventually retaining just significant predictors with a p-value less than or equal to 0.05.

A complete case analysis was used in this study to remove subjects with missing values. A polynomial model was used to test the assumption of linearity for age by introducing a quadratic term. Multicollinearity test for selected individual-level predictors was done to ensure inflated standard errors due to many predictors measuring the same characteristics are controlled. In this research, the parameters for variance inflation factor (VIF) ≤ 2.5 and tolerance ≥0.4 were set as recommended by Johnston et al. [42] for the logistic regression model to identify potentially redundant variables due to collinearity.

Type-3 likelihood ratio test was used to examine categorical explanatory variables that have classifications greater than two. Predictors were considered confounders if the difference in the regression coefficient in the unconditional and conditional model was > 20% [40]. This study tested interactions among predictors that were significant in the multivariable model.

The final model had both fixed and random effects, which were reported as odds ratios and intraclass correlation coefficients (ICC) respectively. To compare the effect on individuals across the communities, this study manually calculated population-averaged odds ratios (ORs) and 95% confidence intervals from the subject-specific coefficients from the final model using the following equation:

Equation 2 Population-averaged Odds Ratios

$$ {\beta}_{\mathrm{PA}}=\beta /\left(\surd \left(1+0.346{\sigma}^2\mathrm{group}\right)\ \right) $$

where σ^2^_group_ is community-level variance, β is the subject-specific regression coefficient.

Based on the latent response variable approach [43], the variance partition coefficient (VPC) which is also referred to as Intraclass Correlation Coefficient (ICC) was calculated for the community in both the null and final model, which measures the variability in the dependent variable attributable to the contextual level [44]. The VPC was computed from this formula below.

Equation 3: Variance Partition Coefficient (VPC)

$$ \mathrm{VPC}={\sigma}^2\mathrm{group}/\Big(\left({\sigma}^2\mathrm{group}+{\pi}^2/3\right)\mathrm{where}\ {\sigma^2}_{\mathrm{group}}\mathrm{is}\ \mathrm{community}-\mathrm{level}\ \mathrm{variance}. $$

This study computed “design effect (deff)”, the quotient of the variance in a clustered data structure relative to that in an independent structure. Due to the fact that the variation within or between clusters for discrete data is not always constant, deff is an approximation [45].

Equation 4: Design effect, deff ≈ 1 + (C - 1) x ICC, where C is average cluster size and ICC represents intraclass correlation coefficient.

The final model in this study was compared with the null model, and a smaller value of Akaike’s Information Criterion (AIC) and Bayesian Information Criterion (BIC) was regarded as a parsimonious model [46]. Also, model diagnostics was done using the area under the curve (AUC) of the receiver-operating characteristic (ROC). Alpha level of 0.05 was used to gauge the association that was statistically significant in this current research. STATA 14 (Stata Corp. Inc., TX, USA) was employed in this study.

## Results

### Descriptive statistics

**Fig. 4:**
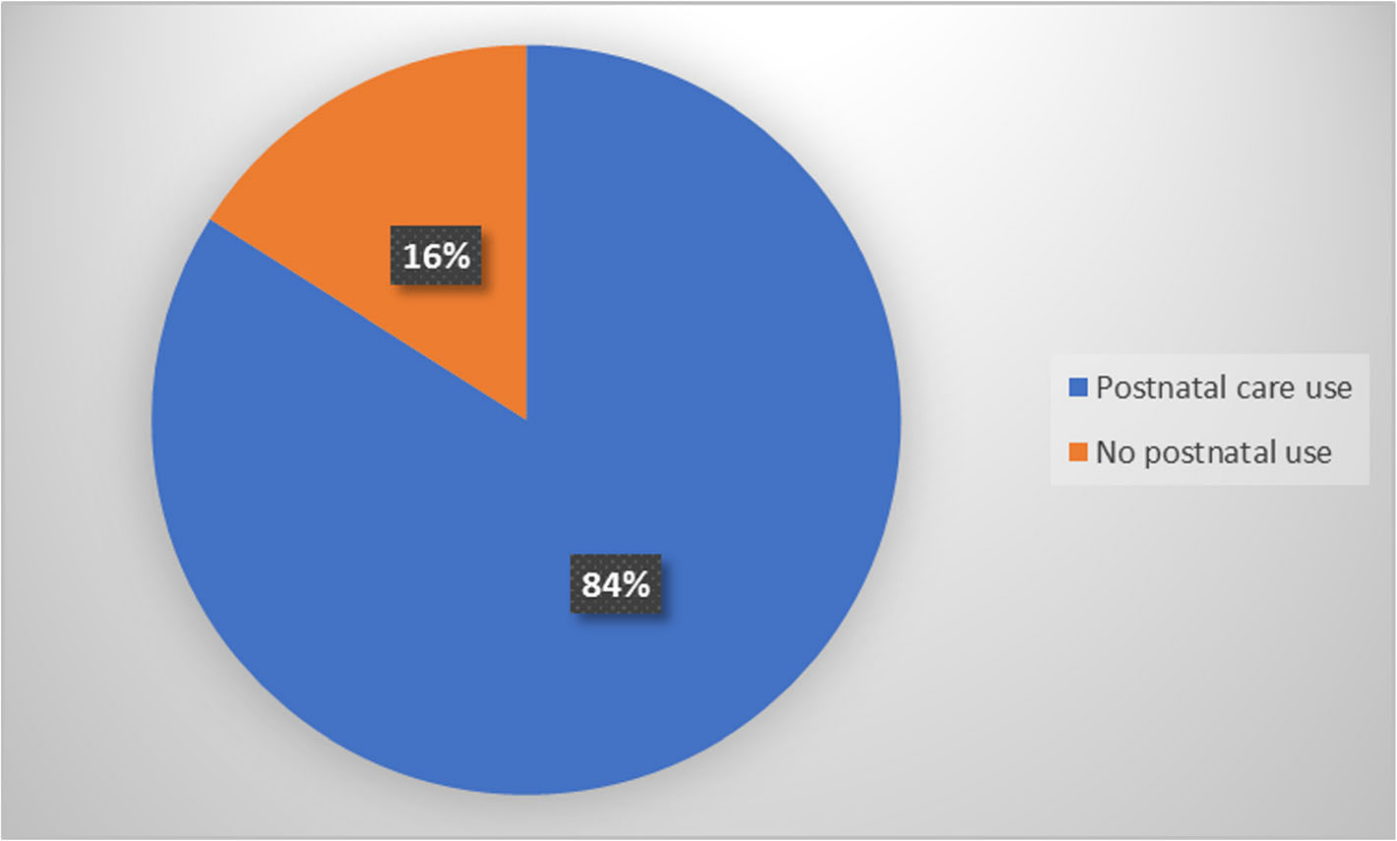
Distribution of postnatal care service utilization among reproductive aged Ghanaian women, 2014 GDHS data

In this study, a total of 427 communities were examined and within all these communities 84% of the women who reported having given live birth within the past 5 years, utilized PNC services whilst 16% did not use with significant inequities in the utilization of this essential service (Fig. 4).

### Spatial autocorrelation

**Fig. 5:**
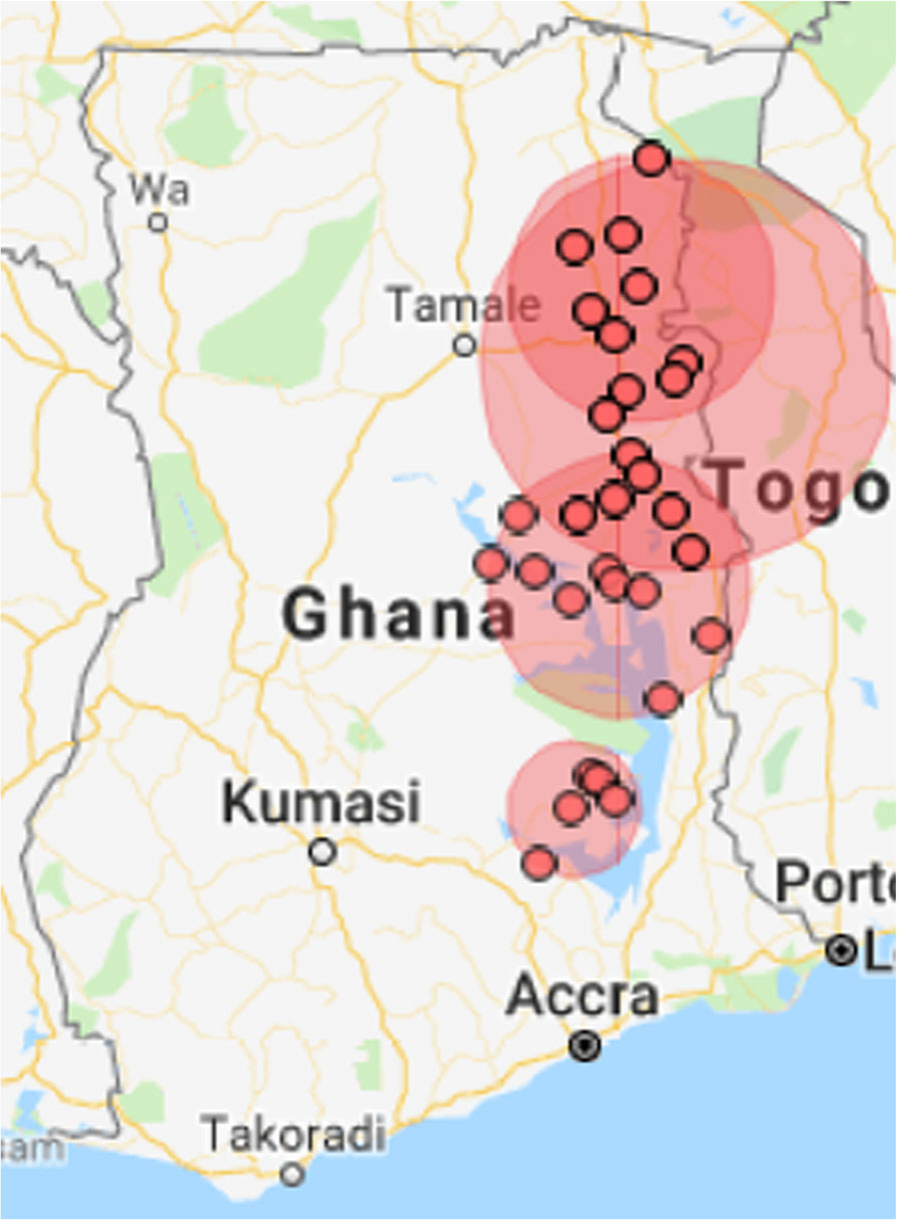
Map of Ghana showing significant clusters of non-utilization of PNC services based on the 2014 Ghana Demographic and Health Survey data (generated by the authors using SaTScan software version 9.6.0 and Google Map)

The median number of study participants per community that reported non-use of PNC services was 1 (IQR: 0–2). Regarding clusters with high rates of non-utilization of PNC services, this study found significant spatial autocorrelations of non-use of PNC services in Ghana (Fig. 5). The spatial scan statistic output identified 4 significant clusters with disproportionately higher non-usage of PNC services than their neighboring communities. The biggest cluster composed of 13 communities in the northern region and three communities in the Volta region, covering a diameter of 123.9 km. This cluster had a relative risk of 3.97 (*p*-value < 0.00001) which indicates that study participants who dwell in the area were 3.97 times more likely to miss PNC services than surrounding communities. Also, a second cluster was detected among ten northern communities covering a diameter of 80.1 km. The risk of not receiving PNC services among respondents who resided in this cluster area was 3.93 times more when compared to those outside that locality. Furthermore, this study detected another significant cluster that comprised of 15 communities: Volta region (8 communities), Brong Ahafo (2 communities) and Northern region (5 communities). The cluster diameter was 78.8 km and the relative risk of not using PNC services in the clustered area was 2.36 (p-value < 0.000003). This revealed that study participants who live in this locality were 2.36 times more likely not to utilize PNC services relative to bordering communities (Fig. 5). Lastly, a cluster was identified in the Eastern region of Ghana which was made up of five communities covering a diameter of 40.78 km; the odds of not using PNC services in this clustered locality had a was 3.51 times higher than respondents in the surrounding communities (Fig. 5).

### Community-level characteristics

The distribution of study predictors by the community has been outlined in Table 2. Concerning the community-level problem with distance to a health facility, 69.3% of the communities did not have a big problem with distance to a health facility while 30.7% had a big problem with distance to a health facility. In terms of PNC services utilization, 87.5 and 76.0% utilization rates were found in communities that did not have a big problem with distance to a health facility and communities with a big problem with distance to a health facility respectively.

**Table 2:** Distribution of women by predictors and PNC services utilization, and unadjusted odds ratios (UOR), 95%CI, and *p*-values for predictors of using PNC services among Ghanaian women from the univariable 2-level logistic regression model

Predictors	Overall (***N*** = 4292)%	Used postnatal care (***N*** = 3605)%	No postnatal care (***N*** = 687)%
**Individual-level characteristics**	**N (%)**	**N (%)**	**N (%)**
Age (Mean **±** SD)	30.9 **±** 7.33	30.6 **±** 7.03	31.3 **±** 7.62
Marital status			
Single	363 (8.5)	317 (87.3)	46 (12.7)
Cohabitating	830 (19.3)	672 (81.0)	158 (19.0)
Widow/divorced/separated	300 (7.0)	254 (84.7)	46 (15.3)
Married	2799 (65.2)	2362 (84.4)	437 (15.6)
Religion			
Traditional/other	324 (7.5)	183 (56.5)	141(43.5)
Islam	922 (21.5)	801(86.9)	121(13.1)
Christians	3046 (71.0)	2621(86.0)	425(14.0)
Ethnicity^b^			
Akan	1642 (38.3)	1473 (89.7)	169 (10.3)
Northern tribes	1795 (41.8)	1411 (78.6)	384 (21.4)
Ewe	476 (11.1)	391 (82.1)	85 (17.9)
Ga	198 (4.6)	179 (90.4)	19 (9.6)
Other	180 (4.2)	150 (83.3)	30 (16.7)
Parity			
One birth	935 (21.8)	826 (88.3)	109 (11.7)
Two births	838 (19.5)	737 (88.0)	101 (12.1)
Three or more births	2519 (58.7)	2042 (81.1)	477 (18.9)
Educational attainment			
No education	1418 (33.0)	1051(74.1)	367 (25.9)
Primary	869 (20.3)	725 (83.4)	144 (16.6)
Secondary/Higher	2005 (46.7)	1829 (91.2)	176 (8.8)
Wealth status			
Poor	2241 (52.2)	1703 (76.0)	538 (24.0)
Middle	811 (18.9)	751 (89.0)	89 (11.0)
Rich	1240 (28.9)	1180 (95.2)	60 (4.8)
Employment status^a^			
Not working	886 (20.7)	760 (85.8)	126 (14.2)
Working	3403 (79.3)	2842 (83.5)	561(16.5)
**Community-level characteristics**			
Community Problem with distance to health facility			
Not a big problem	2973 (69.3)	2603 (87.5)	370 (12.5)
Big problem	1319 (30.7)	1002 (76.0)	317 (24,0)
Area of residence			
Urban	1777 (41.4)	1634 (92.0)	143 (8.0)
Rural	2515 (58.6)	1971(78.4)	544 (21.6)
Community Poverty level			
Low	3046 (71.0)	2720 (89.3)	326 (10.7)
High	1246 (29.0)	885 (71.0)	361 (29.0)
Community Education level			
Percentage of women with at least secondary education per community (Mean **±** SD)	45.1 **±** 26.02	54.6 **±** 26.11	35.6 **±** 26.20
Community unemployment level			
Percentage of women without work per community (Mean **±** SD)	26.4 ± 13.01	27.6 **±** 12.95	25.2 **±** 13.06

N, number of observations; SD, standard deviation; %, percent; ^a^N = 4289 and ^b^N = 4291, due to missing values; CI, confidence in; GDHS data 2014

Regarding area of residence, about 58.6% of the communities were in rural areas whereas about two-fifth (41.6%) of the communities were situated in the urban areas*.* The utilization rate of PNC services in urban communities was 98.0% whilst 78.4% occurred in rural communities (Table 2).

With respect to poverty levels of communities, PNC services utilization rate was 71% in communities with a high poverty level whereas it was 89.3% in communities with a low poverty level (Table 2). This implies that respondents residing in poorer communities have lower utilization of PNC services. The average community-level percentage of respondents with at least secondary education was 45.1 ± 26.02. Community unemployment level variable met the linearity assumption (*p*-value = 0.29). The average percentage of communities’ unemployment was 26.4 ± 13.01 (Table 2).

### Individual-level characteristics

Table 2 displays the distribution of individual-level socioeconomic control variables by PNC services use. Regarding age, the linearity assumption was not violated (p-value = 0.148) and hence was analyzed as a continuous variable. Overall, 4292 study participants were included in the study, their ages were from 15 to 49 years with a mean age of 30.9 ± 7.3 years. Among respondents who received PNC services, the average age was 30.6 ± 7.0 year (Table 2).

Regarding marital status, 65.2% of the study participants were married, 19.3% were cohabitating, 8.5% were single while 7.0% were divorced or widowed or separated. Comparison in the use of PNC services by marital status showed that 87.3% of single respondents received PNC services, 84.7% of divorced or widowed or separated, 84.4% of married and 81.0% of cohabitating study participants used PNC services (Table 2).

Concerning religion, Christians represented 71% of the study respondents. Muslims accounted for 21.5% of women whilst 7.5% were traditional or other believers. In connection with PNC services, 56.5% of women who were traditional or other believers used PNC services. Most Christians (86.0%) and Muslims (86.9%) received PNC services (Table 2).

Northern tribes constituted 41.8% of the study population followed by Akans (38.3%) while other ethnic groupings were the least represented (4.2%) among the study participants corresponding with their percentage in the population. The highest utilization of PNC services was found among Ga respondents (90.4%) whereas northern tribe respondents had the highest non-utilization of PNC services (21.4%)(Table 2).

Considering parity, 58.7% of study participants had at least 3 births whereas 21.8 and 19.5% of the them had given birth once and twice respectively. Further, 81.1% of study participants who had given birth thrice or more used PNC services. Among study respondents who had one birth and two births, 88.3 and 88.0% respectively received PNC services (Table 2).

Concerning education, 33.0% of study participants had no education and 46.7% had at least secondary education. In terms of the use of PNC services, 83.4% of respondents who attained primary education and 91.2% of respondents who had secondary or higher education received PNC services. Among study participants with no education, 74.1% used PNC services (Table 2).

As for wealth status, more than half (52.2%) of the respondents were considered poor, 28.9% were rich, and the remaining18.9% were middle-class. Regarding uptake of PNC services, 76% of poor respondents received PNC service whereas 81.4 and 95.2% of middle-class and rich respondents used PNC services respectively (Table 2). Referring to respondents’ employment status, 79.3% were working and 83.5% of those working received PNC services compared to 85.8% of study participants who were not working (Table 2).

### Inferential statistics

**Univariable analysis results**: Two-level mixed models were fitted to account for community-level variance. Most communities had more than one respondent who participated in the 2014 GDHS, and this has the tendency to cause clustering in the dataset. The number of respondents in the community ranged from 1 to 33 with an average of about 10 study participants per community. The design effect of 4.3 computed in this study suggested a clustered data structure and hence justified the use of multilevel analysis as proposed by Maas and Hox [45]. In the univariate model, only women’s marital (*p*-value = 0.4) and employment (p-value = 0.8) status had *p*-values greater than 0.25 and were excluded from the study (Table 3).

**Table 3:** Distribution of women by predictors and PNC services utilization, and unadjusted odds ratios (UOR), 95%CI, and p-values for predictors of using PNC services among Ghanaian women from the univariable 2-level mixed logistic regression model

Predictors	UOR (95% CI)	P-value
**Individual-level characteristics**		
Age (years)	0.98 (0.97, 0.99)	0.02
Marital status		
Cohabitating	0.84 (0.55, 1.27)	0.4
Widow/divorced/separated	0.94 (0.56, 1.57)	
Married	1.05 (0.72, 1.53)	
Single	Reference	
Religion		
Islam	3.03 (1.98, 4.65)	< 0.0001
Christians	3.04 (2.18, 4.22)	
Traditional/other	Reference	
Ethnicity		
Akan	1.88 (1.26, 2.81)	0.0001
Northern tribes	1.07 (0.71, 1.62)	
Ga	2.69 (1.35, 5.33)	
Other	0.85 (0.49, 1.66)	
Ewe	Reference	
Parity		
Three or more births	0.65 (0.50, 0.85)	0.0005
Two births	0.96 (0.69, 1.33)	
One birth	Reference	
Educational attainment		
No education	0.41 (0.31, 0.52)	< 0.0001
Primary	0.52 (0.40, 0.69)	
Secondary/Higher	Reference	
Wealth status		
Poor	0.18 (0.13, 0.26)	< 0.0001
Middle	0.39 (0.27, 0.58)	
Rich	Reference	
Employment status		
Working	0.97 (0.76, 1.24)	0.8
Not working	Reference	
**Community-level characteristics**		
Community Problem with distance to health facility		
Not a big problem	1.39 (1.11, 1.73)	0.004
Big problem	Reference	
Area of residence		
Urban	3.32 (2.35, 4.68)	< 0.0001
Rural	Reference	
Community Poverty level		
High	0.28 (0.20, 0.41)	< 0.0001
Low	Reference	
Community Education level		
Percentage of women with at least secondary education per community	1.03 (1.02, 1.04)	< 0.0001
Community unemployment level		
Percentage of women without employment per community	1.01 (0.99, 1.02)	0.22

CI, confidence interval; UOR, unadjusted odds ratio; GDHS data 2014

### Multivariable analysis results

Apart from marital status and employment status variables that were not considered in the adjusted model, the rest of the study variables were tested for multicollinearity. All risk factors that were selected for the adjusted model had VIF ≤ 2.5 and tolerance ≥0.4. In addition, the overall mean VIF of 1.58 indicated that multicollinearity was not considered problematic (Table 4).

**Table 4:** Results of multicollinearity test for selected predictors for the multivariable model

Predictors	VIF	Tolerance
**Individual-level characteristics**		
Age	1.66	0.60
Religion	1.21	0.83
Ethnicity	1.03	0.97
Parity	1.73	0.58
Education	1.86	0.54
Wealth status	2.42	0.41
**Community-level characteristics**		
Community Problem with distance to health facility	1.33	0.75
Area of residence	1.82	0.55
Community Education level	2.50	0.40
Community Poverty level	1.52	0.66
Community unemployment level	1.05	0.95
**Mean VIF**	**1.58**	

**Fig. 6:**
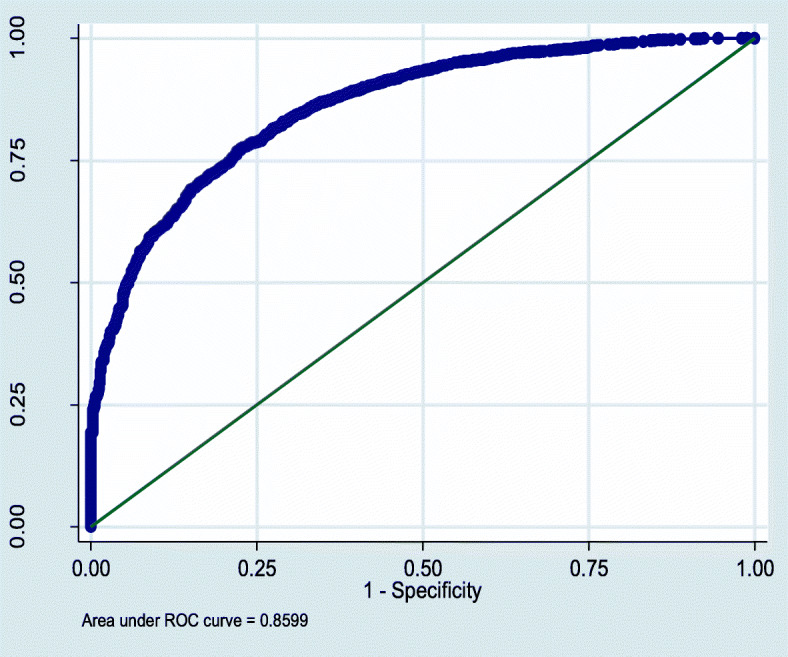
Receiver Operating Characteristics curve of the final model

As shown in Table 5, the final model had smaller AIC and BIC; therefore, was selected as a more parsimonious model (AIC = 3146.7; BIC = 3235.8) than the model without risk factors (AIC = 3304.9, BIC = 3317.6). The AUC of 0.86 (95%CI: 0.85–0.87) from the ROC curve demonstrates the model is a good binary classifier of PNC service use or not (Fig. 6).

**Table 5:** Population-averaged Odds ratios (OR) and 95%CI for socioeconomic predictors of using PNC services among Ghanaian women from the final 2-level mixed logistic regression model

Predictors	Null model OR (95% CI)	Final model AOR (95% CI)	P-value
**Fixed effect**			
**Individual-level characteristics**			
Age (years)		1.00 (0.98, 1.01)	0.9
Religion			
Muslim		2.42 (1.68, 3.49)*	< 0.0001
Christians		1.99 (1.50, 2.63)*	< 0.0001
Traditional/other		Reference	
Ethnicity			
Akan		1.46 (1.05, 2.05)*	0.03
Northern tribes		1.74 (1.19, 2.54)*	0.004
Ga		1.87 (1.05, 3.33)*	0.03
Other		1.05 (0.62, 1.77)	0.9
Ewe		Reference	
Educational attainment			
No education		0.72 (0.56, 0.92)*	0.009
Primary		0.60 (0.61, 0.99)*	0.04
Secondary/Higher		Reference	
Parity			
Three or more births		0.87 (0.69, 1.11)	0.3
Two births		1.01 (0.76, 1.35)	0.9
One birth		Reference	
Wealth status			
Poor		0.44 (0.31, 0.63)*	< 0.0001
Middle		0.60 (0.43, 0.85)*	0.004
Rich		Reference	
**Community-level characteristics**			
Community Problem with distance to health facility			
Not a big problem		1.08 (0.89, 1.32)	0.4
Big problem		Reference	
Area of residence			
Urban		1.05 (0.75, 1.46)	0.8
Rural		Reference	
Community Poverty level			
High		0.60 (0.44, 0.81)*	0.001
Low		Reference	
Community Education level			
Percentage of women with at least secondary education per community		1.01 (1.01, 1.02)*	0.001
Community unemployment level			
Percentage of women without employment per community		0.99 (0.98, 1.00)	0.5
**Random effects**	**Null model**	**Final model**	
Community level variance (95% CI))	1.84** (1.40, 2.42)	1.02 (0.73, 1.42)*	
ICC (95% CI)	0.36 (0.30, 0.42)	0.24 (0.18, 0.30)*	
**Model fit statistics**			
AIC	3304.9	3146.7	
BIC	3317.7	3235.8	

*CI* confidence interval, *OR* Odds ratio, *SE* Standard error, *ICC* Intraclass Correlation Coefficient, *AIC* Akaike Information Criterion, *BIC* Bayesian Information Criterion; *significant at *p* < 0.05; GDHS data 2014

The results from the multivariable 2-level mixed model with logit link function are shown in Table 5. In this study, potential confounders were maintained in the model. No interaction term was found beyond the significant main effects of the predictors in the multivariable model.

### Community-level effect (random effects)

In this study, intraclass correlation coefficients (ICC) computed from the 2-level mixed logistic regression models were used to measure the degree of heterogeneity in PNC services use accounted for by the differences between communities. The ICCs showed significant proportions of variability in PNC utilization in this study. The intercept only model (without study variables) examined in this study reported significant variation in PNC services utilization across the communities (ICC =0.36 (95% CI: 0.30–0.42). The variation in the use of PNC services across the community continued to be significant after including individual-level study predictors. The result from the final model showed that 24% (95% CI: 0.18–0.30) of the unobserved variation in PNC services utilization could be explained by community heterogeneity (Table 5).

Moreover, the results of the association between community-level predictors and receiving PNC services are shown in Table 5. This analysis indicates that the odds of using PNC services by a woman, who resided in a community with a higher poverty level was 0.60 (95%CI:0.44–0.81) times lower than a woman who lived in a community with a lower poverty level. Also, a moderate association between community-level secondary or higher education and the use of PNC services was identified. For a one percentage change in the level of community secondary or higher education, the odds of using PNC services increases by 1.01 (95%CI:1.01–1.02) times. However, the analyses indicate that the effect of a community-level problem with distance to a health facility, area of residence and community-level unemployment on the utilization of PNC services in the crude model were diminished in the multivariable model after controlling for other explanatory factors. The use of PNC services did not have an independently significant association with community problem with distance to a health facility (AOR = 1.08, 95%CI: 0.89, 1.32), area of residence (AOR = 1.05, 95%CI: 0.75–1.46) and community-level unemployment (AOR = 0.99, 95%CI: 0.98–1.00).

### Individual-level effect

Based on Table 5, the findings of this study show that religion, ethnicity, education, and wealth status emerged as significantly associated with services uptake. The study results showed that Muslims (AOR = 2.42, 95%CI: 1.68–3.49) and Christians (AOR = 1.99, 95%CI: 1.50–2.63) were more likely to receive PNC services compared to women who were traditional and other believers from any community. Similarly, odds of receiving PNC services by women who were Akan (AOR = 1.46, 95% CI: 1.05–2.05), Northern tribes (AOR = 1.74, 95%CI: 1.19–2.54), and Ga (AOR = 1.87, 95%CI:1.05–3.33) were higher than Ewe women from any community. Also, the findings from this research highlighted that the odds of receiving PNC services among women with no education (AOR = 0.72, 95%CI: 0.56–0.92) and primary educated women (AOR = 0.60, 95%CI: 0.61–0.99) were lower when compared to women who attained secondary or higher education from any community. On wealth status, the odds of receiving PNC services among poor and middle-class women were 0.44 (95%CI: 0.31–0.63) and 0.60 (95% CI: 0.43–0.85) times respectively lower than rich women who reside in any community. Conversely, individual-level factors such as age, and parity were not significantly associated with the use of PNC services (Table 5).

## Discussion

This study reported a higher utilization rate of PNC services across communities than findings of the Sakeah study team in Ghana; this discrepancy may have arisen because they studied only two rural districts with a smaller sample size [12].

Also, this research found that the community-level variables: problems with distance to a health facility; area of residence; and community-level unemployment were not significantly associated with the use of PNC services. In agreement with this research, Mohan et al. [23] reported no significant association between community-level distance to a health facility and PNC services utilization. Also similar to this study, some researchers reported that the association between use of PNC services and area of residence was not significant [25, 33]. In addition, a study by Darega et al. [47] found no significant association between employment status and the use of PNC services. However, the results of this study are not consistent with other studies conducted in other developing countries which found a significant association between the use of PNC services and employment status [13]. This might be explained by the fact that contrary to these previous studies, which were carried out at the individual level, this study assessed community-level unemployment independent of individual level characteristics so there is a difference in the level of analysis.

Most importantly, this study found significant variability in use of PNC services utilization at the community level independent of individual-level characteristics. The association between community-level poverty and use of obstetric care services has long been established in the literature [34, 47–49]. Nonetheless, the evidence is somewhat mixed as the results from a previous study that was conducted in the rural part of Tanzania found no substantial association between community-level poverty and utilization of PNC services [23]. This current study adds to the body of evidence indicating that poverty at the community level matters over and above poverty at the individual level; this finding may be explained by women in poorer communities inability to afford indirect costs such as transportation, illegal fees being demanded at health facilities among other costs that are often required for accessing obstetric services [50], even though postdelivery care itself is free. Also, women from poorer communities might suffer discrimination from health workers [51, 52], which could make them avoid further contact with the health system including PNC services. Lastly, as expected evidence confirms that women from richer communities face lower barriers to receiving needed maternal healthcare [53].

Contrary to a study that found no significant effect of community-level education on the utilization of PNC services [23, 49], this research identified a significant association between the use of postnatal services and community-level education. The limited number of explanatory variables that were used in the previous studies could explain the discrepancies in the findings because confounding variables can potentially alter the results when they are insufficiently controlled. The findings of this study highlight the importance of community-level education, in that higher education could mean better access to health information and hence better understanding of the benefits of maternity services, and often more autonomy to choose evidence-based obstetric services rather than some potentially harmful cultural practices [54].

Additionally, this study identified that most of the significant clusters of non-utilization of PNC services were found in the Northern region. This result affirms the long-held notion of spatial disparities in the utilization of obstetric care services between northern and southern regions of Ghana [48, 55]. Similar trends have been observed by other studies on maternal health services utilization [51, 52]. Also, this study revealed that a significant portion of the variation in PNC services use was attributed to unobserved community-level variance like other studies elsewhere [23, 49]. Specifically, this study found that about 24% of variability in the usage of PNC services could be explained by unmeasured community-level characteristics.

Apart from women’s community poverty and educational level that may partially explain the inequalities in the use of PNC services as identified in this study, this spatial and unexplained variation in the utilization of PNC could be as a result of some potentially relevant community-level factors for which data are not available. The geographical variation in the non-utilization PNC services may be attributed to inadequate number of health facilities and health professionals in the northern region as suggested the earlier Addai study [55]. Also, media exposure tends to influence the community’s uptake of maternal health services including PNC services based on previous studies [24, 34]. The differential usage of PNC services perhaps may be due to the influential role of cultural values and practices as reported in other studies [56]. However, the GDHS data lacks variables which could be used as proxies for cultural variables that influence maternal health-seeking behavior, and so further studies are required to unravel the influence of community’s cultural underpinnings on the use of PNC services. Future research is warranted to identify further community-level characteristics that could explain the unmeasured community heterogeneity in PNC services uptake in Ghana. Notwithstanding, the link between the community where women reside, and the use of PNC services established in this study is of importance since it provides insight into the community-level influence to help address the inequalities in PNC services use.

Concerning individual-level predictors, some past studies did not find significant association between PNC services utilization and women’s age [23, 49], marital status [25], employment status [14, 15], and parity [23] similar to the findings of this present study. In this current research, wealth status of women was found to be significantly associated with the use of PNC services. The odds of receiving PNC services were lower among the poor and middle-class women than rich women. This result is consistent with findings of studies conducted in Nigeria [20], India [28] and Bangladesh [57]. The literature seems to unequivocally suggest that wealthier women have better access to maternal health services in general since they can afford the ancillary costs that are related to accessing PNC services [58].

Also, some comparable studies in other developing countries reported that maternal education significantly influences PNC service utilization [20, 23, 59]. Consistent with these studies, this research found that lower education was negatively associated with uptake of PNC services. Specifically, this study identified lower odds of using of PNC services among women with no education and women with primary education relative to women with at least secondary education. This is an expected finding and can be explained by an educated women’s higher likelihood to be more informed about health risks and benefits which is then translated into demand for PNC services [11]. On the other hand, less educated women may not be knowledgeable about availability and accessibility of PNC services as well as how the health system operates [49]. Also, these lower educated women may have less say in decision-making about their health and this eventually affects PNC services uptake [59].

Moreover, this research revealed that religion is significantly associated with the use of PNC services with traditional and other believers having significantly lower levels of PNC utilization, which is consistent with the findings of a study by Ononokpono et al. [20]. This study finding may be explained by previous research’s findings that reported on the traditional cultural practice of keeping newborns from the public due to fear of harm in the first month after their birth [56]. To increase the uptake of PNC services among this group, the involvement of religious leaders and home visits have been proposed by earlier studies [56, 60]. This study suggests that the effect of religion on postnatal care use should be further investigated to better understand the underpinnings.

Finally, some sub Saharan African studies are consistent with this research that ethnicity is a significant predictor of PNC services utilization [20, 61]. Ethnic groups are predominantly made up of people who share similar characteristics, and this has the potential to influence their perception about health and ultimately women’s health seeking behavior [20, 62].

One of the key goals of this study is to generate evidence that could be used to inform equity-based interventions. The conceptual framework for action on the social determinants of health proposed by the WHO Commission on Social Determinants of Health (CSDH) offers useful insights on addressing health inequities [63]. This framework considers the health system itself an intermediary determinant of health and hence exploring factors influencing access to health services is key to understanding and addressing health inequities. Our study indicates that the social determinants play–both at the individual and community levels- are key to addressing inequity in access to health care services, and inequities in health in turn.

### Study strengths and limitations

This study contributes to the growing literature on the effect of community-level factors on the uptake of PNC services. Multilevel mixed modeling was used to ascertain the impact of community-level factors on the use of PNC services, which is a more advanced approach to estimating the relationships than much of the available literature. Also, this study highlighted communities with a higher risk of not receiving PNC services for targeted interventions. Another significant strength of this research is the use of a large nationwide population-based dataset with a very high (97%) response rate. Despite these strengths, there are limitations in interpreting the results. First, self-reported data can lead to information bias, which could affect accurate classification. Recall bias could be a concern; however, information on the women’s PNC services use was restricted to 5 years preceding the survey and hence recall bias is probably not a major concern in this study. Also, medical need and information on quality of service received as well as cultural variables were not available in the secondary dataset of the 2014 GDHS. This study considered enumeration areas as communities, which may not necessary be representative of the actual communities because they have arbitrary boundaries. The 2014 GDHS lacked variables to examine the effect of travel time, quality of care, transportation system and cost of travel on the use of obstetric care services [24]. For this reason, only distance to a health facility was used in this research; relying on the response to the question of whether the distance to a health facility was either a “big problem or not a big problem” in the survey. Also, the GPS points taken in the 2014 GDHS were deliberately displaced randomly at a maximum of 2 km and 5 km in urban and rural communities respectively and seldom repositioning at random one GPS point by 12 km to ensure confidentiality and to conceal the identity of the respondents; so it is not probable that the findings from this study would be affected. Lastly, causality of the association cannot be inferred because a cross-sectional study design was used.

## Conclusions

This study adds to the growing body of literature on the social determinants of health and our findings clearly point in the same direction as the CSDH framework for action on the social determinants of health. This framework asserts that addressing the structural upstream determinants of health, including inequities rooted in factors such as socioeconomic position, social class, education, and income is the most effective and sustainable approach to reducing health inequities. However, it is hardly surprising that it is much harder to address these upstream factors and hence, many of the interventions (action) to address inequities in health have focused on changing behaviors and not the roots of these health-seeking behaviors.

Based on our findings and consistent with the CSDH framework for action on the social determinants of health, factors such as income and education- both at the individual and community level- cannot be ignored if we hope to achieve real progress on improving equity in access to PNC services. Governments could choose different universal or targeted approaches to addressing inequities. Our findings regarding community level variation and the effect of community level factors indicate that it may not be sufficient to target subsidies to economically disadvantages women and tailor health messaging to women with lower education, community-level interventions are needed. Moreover, we identified hotspots of non-use of PNC services, concentrated in the Northern region that could be identified as priority intervention areas.

The findings also highlight the need to better understand the role of culture and religious beliefs in influencing access to PNC services; it is not possible to consider interventions to improve equity related to these factors without an in-depth understanding of factors using qualitative research. In addition, community-level variation also needs to be understood better in order to devise effective strategies to address community-based health inequities.

## Data Availability

The study dataset is available in the MEASURE DHS repository, https://www.dhsprogram.com/data/dataset_admin/login_main.cfm. ^[GHIR72DT]^

## Abbreviations

AIC: Akaike’s Information Criterion
AOR: Adjusted odds risk
AUC: Area under the curve
BIC: Bayesian Information criterion
CI: Confidence Interval
CSDH: Commission on Social Determinants of Health
GDHS: Ghana Demographic and Health Survey
GPS: Global positioning system
GSS: Ghana Statistical Service
ICC: Intraclass correlation coefficient
OR: Odds Ratio
PA: Population average
PNC: Postnatal care
ROC: Receiver operating characteristics
SD: Standard deviation
SE: Standard error
UOR: Unadjusted odds ratio
VIF: Variance Inflation Factor
VPC: Variance partition coefficient.
